# Risk factors associated with the prevalence of neck and shoulder pain among high school students: a cross-sectional survey in China

**DOI:** 10.1186/s12891-023-06656-8

**Published:** 2023-08-09

**Authors:** Ruilong Wang, Yue Yin, Haoliang Zhang, Letian Pan, Yuanting Zhu, Mingxiu Wang, Ziyin Huang, Weiheng Wang, Guoying Deng

**Affiliations:** 1 Trauma Center, Shanghai General Hospital, Shanghai Jiao Tong University School of Medicine, Shanghai, 200080 China; 2 Present Address: Shanghai General Hospital, Shanghai Jiao Tong University School of Medicine, No. 650 Xin Songjiang Road, Shanghai, 201620 China; 3 Department of Laboratory Medicine, Shanghai General Hospital, Shanghai Jiao Tong University School of Medicine, Shanghai, 200080 China; 4 Department of Orthopaedics, Second Affiliated Hospital of Naval Medical University, NO.415 Fengyang Road, Shanghai, China

**Keywords:** High school students, Neck and shoulder pain, Demographic factors, Lifestyle habits, Risk factors

## Abstract

**Background:**

After the COVID-19 outbreak, many Chinese high school students have increased their dependence on electronic devices for studying and life, which may affect the incidence of neck and shoulder pain (NSP) in Chinese adolescents.

**Methods:**

To evaluate the prevalence of NSP in high school students and its associated risk factors during COVID-19, a survey was conducted among 5,046 high school students in Shanghai, Qinghai, Henan and Macao during the second semester and summer vacation of the 2019–2020 academic year. The questionnaire included questions regarding demographic characteristics, the prevalence of NSP and lifestyle factors such as sedentary behavior, poor posture and electronic device usage. Univariable and multivariable logistic regression was used to analyze the possible influencing factors for neck and shoulder pain.

**Results:**

A total of 4793 valid questionnaires (95.0%) were collected. The results indicated that the prevalence of NSP was 23.7% among high school students. Binary logistic regression analysis revealed that female gender (P < 0.05, OR = 1.82), grade (P < 0.05, range OR 1.40–1.51) and subject selection (P < 0.05, range OR 0.49–0.68) were risk factors for NSP in high school students. Sedentary behavior (P < 0.05, range OR 1.74–2.36), poor posture (P < 0.05, range OR 1.19–2.56), backpack weight (P < 0.05, range OR 1.17–1.88), exercise style and frequency (P < 0.05, range OR 1.18–1.31; P < 0.05, range OR 0.76–0.79, respectively), and the time spent using electronic devices (P < 0.05, range OR 1.23–1.38)had a significant correlation with NSP in high school students.

**Conclusions:**

NSP is currently very common among high school students during the outbreak of COVID-19. Sedentary behavior, poor posture and other factors have a great impact on the occurrence of NSP in high school students. Education regarding healthy lifestyle choices should be advocated for to decrease NSP among high school students, such as more physical activity, changing poor postures and reducing the amount of time spent using electronic devices.

## Introduction

Neck and shoulder pain (NSP) is caused by a group of diseases involving dysfunction of the cervical spine, nerve or soft tissue [1]. The prevalence of NSP in the adult population has been as high as 86.8%, which seriously affects people’s health and quality of life [2]. With changes in lifestyle and the increasing pressures of work, the younger generation is more susceptible to NSP. A survey from Europe showed that 26.1% of adolescents experienced NSP at least once a week [3]. Some studies have indicated that the prevalence of NSP in adolescents is increasing in some developing countries [4]. Studies have shown that NSP is more harmful to teenagers and can lead to serious consequences [5]. NSP should be given more attention; otherwise, it will gradually worsen and develop into a more serious chronic disease [6].

Currently, due to the heavy burden of education, many Chinese high school students do not get enough exercise, and the increasing dependence on electronic devices for studying and life exacerbates this trend [7–9]. During the COVID-19 outbreak, due to the increasing dependence on electronic products and decreased time spend engaging in outdoor activities, the styles of learning and daily behaviors of high school students has possibly changed, which may significantly affect the incidence of NSP [10–12]. Despite the significant lifestyle changes that high school students have undergone during COVID-19, there remains a dearth of relevant research that examines the altered risk factors of NSP among this vulnerable population. Consequently, there is a pressing need to develop and implement more precise, timely, and comprehensive intervention measures that are specifically targeted at preventing NSP among high school students.

Therefore, this study will systematically investigate the prevalence and risk factors for NSP among high school students in some regions of China, especially the main influencing factors relevant to NSP during the COVID-19 epidemic. This investigation will provide consultation for the scientific prevention of NSP for current teenagers.

## Methods

### Study design

This cross-sectional study was designed to investigate the risk factors associated with the prevalence of NSP among high school students. From July 2020 to August 2020, a total of 5,046 students were selected from Shanghai, Qinghai Province, Henan Province and Macao Special Administrative Region (representing eastern, western and central parts of China, Hong Kong, Macao and Taiwan, respectively) according to a cluster randomization method. A total of 4793 valid questionnaires were collected, for an effective rate of 95.0% (4793/5046).

### Ethical approval

All of the participants (if subjects are under 16, from a parent and/or legal guardian) involved in the study provided their electronic written informed consent before being surveyed. This study followed the Helsinki Declaration. The study protocol was reviewed and approved by the Ethics Committee of Shanghai General Hospital, Shanghai Jiao Tong University, School of Medicine (Approval No. 2013KY002).

### Exclusion criteria

Recent injury (less than one month) or deformity of the neck or shoulder, diagnosis of mental illness, and a history of NSP with an obvious cause were excluded. To ensure the validity of the data, questionnaires with incomplete answers, apparent errors unrelated to the question or answer options, and obvious errors in logic were excluded before the analysis.

### Questionnaire design

The questionnaire was designed based on previous research methods and preliminary investigations from the relevant literature [11, 13]. The questionnaire was divided into three parts to analyze the prevalence and influencing factors of NSP among high school students. The first part included demographic information such as gender, grade, school location and subject selection. The second part was the respondents’ awareness of the symptoms of NSP. Interviewees were asked questions regarding the frequency and degree of neck discomfort they experienced to judge whether they suffered from NSP. Students with severe symptoms were advised to go to a nearby hospital. The third part investigated the lifestyle factors associated with NSP. The questions were mainly categorized into exercise preference and frequency, sedentary time and posture, poor posture, backpack weight, and the time spent and habits of the students when using electronic devices before and after online classes.

The respondents were trained to give lectures about several concepts in the questionnaire to avoid inducing bias. A pain attack was defined as a relatively mild pain (scoring 1–4 by Numerical Rating Scale (NRS)) lasting for more than 10 minutes, and “neck and shoulder pain” was defined as “(i) pain over the neck/shoulder region for more than 6 hours each episode or (ii) short-term pain with high frequency more than 2–3 times every day, and (iii) this situation happened more than 3 times in one month”. Instead of directly using “yes” and “no” to assess the exposure to risk factors, the onset frequency of pain was classified into the following four levels: “almost never”, less than once per month; “occasionally”, 1–3 times per month; “often”, 1–3 times per week; and “always”, more than 3 times per week. General interpretation of the results was as follows: “often” and “always” were treated as “yes”, while the other two levels, “almost never” and “occasionally”, were treated as “No”. “Poor posture” was defined as maintaining the same posture for a duration surpassing 1 hour over a period extending beyond three months, which perpetuates the tension and contraction of the muscles, such as crossed legs, hunched walking and the neck being in a forward posture. Sedentary was defined as continuous sitting for 2 hours a day.

### Data analysis

The survey data were extracted and analyzed anonymously by Statistical Package for Social Science software (Version 21.0, SPSS Inc., Chicago, IL, USA). Multiple logistic regression was used, FDR (false discovery rate) was applied to adjust the p-value, and a cross-table was made to explore the correlation between factors and the incidence of NSP in high school students. Questionnaires with incomplete answers, apparent errors unrelated to the question or answer options, and obvious errors in logic were excluded before the analysis. Multiple logistic regression analysis was used to examine all the risk factors, and those with p values < 0.05 were extracted. A backward stepwise regression procedure was performed, and the threshold for variant removal was set at 0.05. The results were represented by odds ratios (ORs) and 95% confidence intervals (CIs), and statistical significance was set at p < 0.05 on logistic regression.

## Results

In this survey, a total of 5,200 questionnaires were delivered, and 5,046 were collected for this study. Incomplete questionnaires or those with obvious logical errors in the completion results were excluded. Ultimately, 4794 valid questionnaires were obtained. The effective recovery rate was 95%.

### Demographic information and NSP prevalence

The demographic characteristics of the participants are shown in Table 1. The total prevalence of NSP in high school students was 23.7%. The respondents comprised 2234 males and 2559 females, and the proportion of NSP students was 18.1% (404/2234) and 28.6% (732/2559), respectively. The results showed that the prevalence of NSP in females was significantly higher than that in males (P < 0.001, OR = 1.815). Regarding subject selection, the number of students majoring in liberal arts and science was 815 (30.3%) and 3192 (22.8%), respectively, and students majoring in liberal arts had a higher prevalence of NSP than students majoring in science (P < 0.001, OR = 1.473). The number of students in grade one, grade two and grade three was 1831 (38.2%), 1418 (29.6%) and 1544 (32.2%), respectively. The prevalence of NSP in grade one was 19.7% and in grade two and grade three was 27.0% and 25.5%, respectively. The prevalence of NSP in grade two and grade three students was significantly higher than that in grade one (P < 0.001, OR = 1.512; P < 0.001, OR = 1.395, respectively). The difference between grade two and grade three was not significant (P > 0.05).

**Table 1:** Demographic factors of neck and shoulder pain

Project	N	Prevalence (%)	p value	OR (96%CI)
**Gender**				
male	2234 (46.6%)	18.1%		1
female	2559 (53.4%)	28.6%	< 0.001	1.815 (1.581 ~ 2.083)
**Grade**				
one	1831 (38.2%)	19.7%		1
two	1418 (29.6%)	27.0%	< 0.001	1.512 (1.283 ~ 1.782)
three	1544 (32.2%)	25.5%	< 0.001	1.395 (1.186 ~ 1.641)
**Subject selection**				
liberal arts	815 (17.0%)	30.3%		1
science	3192 (66.6%)	22.8%	< 0.001	0.679 (0.573 ~ 0.806)
other	786 (16.4%)	20.5%	< 0.001	0.592 (0.471 ~ 0.745)

### Backpack weight and NSP prevalence

Backpack weight is a risk factor for NSP. The data showed that the prevalence and the OR values of NSP among adolescents were in direct proportion to the weight of school backpacks (Table 2).

**Table 2:** Neck and shoulder pain and backpack weight

Project	N	Prevalence (%)	p value	OR (96%CI)
0–2 kg	1310 (27.3%)	20.2%		1
2–4 kg	1701 (35.5%)	22.5%	< 0.001	1.168 (0.978 1.395)
4–6 kg	1094 (22.8%)	26.2%	0.001	1.393 (1.149 1.688)
6–8 kg	381 (7.9%)	26.2%	0.027	1.351 (1.034 ~ 1.766)
> 8 kg	307 (6.4%)	33.2%	< 0.001	1.884 (1.428 ~ 2.485)

### Poor postures and NSP prevalence

Poor postures included sitting with crossed legs, walking with a hunchback, holding neck forward, rounding the shoulders and using a one-shoulder backpack in this investigation. Our study showed that the OR values of sitting with crossed legs, walking with a hunchback, holding neck forward, rounding the shoulders and using a one-shoulder backpack were all greater than 1.0, and the OR value of holding the neck forward was 1.800, indicating that all the poor postures, especially neck forward, were in direct proportion to NSP and that these were risk factors for NSP (P < 0.05). Overall, high school students with at least one poor posture, including sitting with crossed legs, walking with a hunched back, leaning neck forward, having round shoulders or carrying a backpack, were more likely to have NSP than students without any of these poor postures (P < 0.001, OR = 2.555) (Table 3).

**Table 3:** Neck and shoulder pain and poor postures

Project	N	Prevalence (%)	p value	OR (96%CI)
Crossed legs	2700 (56.3%)	26.6%	0.036	1.186 (1.012 ~ 1.391)
Walking hunchback	1856 (38.7%)	30.3%	< 0.001	1.339 (1.155 ~ 1.553)
Neck forward	1204 (25.1%)	36.2%	< 0.001	1.800 (1.540 ~ 2.104)
Round shoulder	727 (15.2%)	37.8%	< 0.001	1.631 (1.364 ~ 1.950)
Single shoulder backpack	918 (19.2%)	30.0%	0.018	1.224 (1.035 ~ 1.449)
Have at least one of these posture problems	3875 (80.8%)	26.4%	< 0.001	2.555 (2.072 ~ 3.151)
All have no	918 (19.2%)	12.3%	< 0.001	0.444 (0.373 ~ 0.529)

### Exercise and NSP prevalence

Exercise habits mainly include strenuous, moderate and easy sports. Strenuous sports include ball games and competitive sports, moderate sports include long-distance running, and easy sports include walking slowly. The data showed that it was helpful to reduce the incidence of NSP by performing more intense exercise. Moderate to low-intensity exercise had relatively little effect on reducing the prevalence of NSP, while the data showed no significant difference (P > 0.05) (Table 4).

**Table 4:** Neck and shoulder pain and exercise habits and frequency

Project	N	Prevalence (%)	p value	OR (96%CI)	
**Exercise habits**					
strenuous	1568 (32.7%)	21.0%		1	
moderate	1103 (23.0%)	23.8%	0.082	1.178 (0.979 ~ 1.416)	
easy	2122 (44.3%)	25.7%	0.001	1.310 (1.121 ~ 1.531)	
**Exercise frequency**					
2 ~ 3 times a month	2411 (50.3%)	26.0%		1	
1 ~ 2 times a week	1911 (39.9%)	21.6%	0.001	0.785 (0.681 ~ 0.905)	
Every day	471 (9.8%)	21.0%	0.025	0.760 (0.598 ~ 0.966)	

In addition, the proportion among students who exercised 2 or 3 times a month, 1 or 2 times a week and every day was 50.3% (2411/4793), 39.9% (1911/4793) and 9.8% (471/4793), and the NSP prevalence was 26.0%, 21.6% and 21.0%, respectively. The data showed a negative correlation between an increased frequency of exercise and the prevalence of NSP (P < 0.05). High school students who were active in exercise generally had a lower risk of NSP than those who were not active (Table 4).

### Electronic device usage and NSP prevalence

Participants who often used electronic devices tended to suffer from NSP (Tables 5 and 6). The amount of time high school students spent using various electronic devices increased significantly during the online semester due to the influence of the epidemic. The data showed that students who spent more than 8 h a day using electronic devices (P < 0.05, OR = 1.230) had a higher prevalence of NSP than those who spent less than 4 h a day. The prevalence of NSP was positively correlated with the amount of time spent using various electronic devices. Computer usage led to a higher prevalence of NSP than other handheld electronic devices when used for the same amount of time (P < 0.05) (Table 5).

**Table 5:** Neck and shoulder pain and time spent using electronic devices

Project	N	Prevalence (%)	p value	OR (96%CI)
**Time spent on hand-held electronic devices**				
0–4 h per day	1431 (29.9%)	17.7%		1
4–8 h a day	1986 (41.4%)	25.9%	< 0.001	1.260 (1.046 ~ 1.519)
More than 8 h a day	1376 (28.7%)	26.9%	< 0.001	1.230 (0.992 ~ 1.525)
**Computer Time**				
0–4 h per day	2682 (56.0%)	19.6%		1
4–8 h a day	1346 (28.1%)	28.5%	< 0.001	1.365 (1.144 ~ 1.629)
More than 8 h a day	765 (16.0%)	29.5%	< 0.001	1.376 (1.087 ~ 1.740)

**Table 6:** Neck and shoulder pain and posture for using electronic devices

Project	N	Prevalence (%)	p value	OR (96%CI)
Standing posture using electronic				
Stood	771 (16.1%)	27.0%		1
Bow head	3596 (75.0%)	24.3%	< 0.001	0.901 (0.751 ~ 1.081)
Facing straight	426 (8.9%)	12.4%	0.004	0.474 (0.334 ~ 0.672)
Sitting posture using electronic				
Stoop	1949 (40.7%)	26.0%		1
Bow head	2312 (48.2%)	23.8%	< 0.001	0.913 (0.790 ~ 1.055)
Facing straight	532 (11.1%)	14.8%	< 0.001	0.628 (0.476 ~ 0.829)

In addition to the amount of time spent using electronic devices, students’ postures also had an impact on the prevalence of NSP. When using a standing posture, the data showed that students who used electronic devices while directly facing the device had a significantly lower prevalence of NSP than those who used electronic devices while stooping(P < 0.05, OR = 0.474). When sitting down and using electronic devices, students who used electronic devices while facing straight had a lower prevalence of NSP than students who used electronic devices while stooping (P < 0.05, OR = 0.628) (Table 6).

Sedentary time was associated with NSP. According to our research data, 23.0% of the respondents were occasionally sedentary, with 15.0% suffering from NSP. A total of 40.6% were often sedentary, with 23.5% suffering from NSP. A total of 36.4% were always sedentary, with 29.4% suffering from neck and shoulder pain. Sedentary frequency was in direct proportion to the risk of NSP, and students who were always sedentary had a higher prevalence of NSP than students who were occasionally sedentary (P < 0.05, OR = 2.360) (Table 7).

**Table 7:** Neck and shoulder pain and sedentary behavior

Project	N	Prevalence (%)	p value	OR (96%CI)
**Sedentary frequency**				
occasionally	1100 (23.0%)	15.0%		1
often	1945 (40.6%)	23.5%	< 0.001	1.740 (1.431 ~ 2.117)
always	1748 (36.4%)	29.4%	< 0.001	2.360 (1.942 ~ 2.868)

## Discussion

Previous studies have shown that NSP has a high prevalence and can have serious consequences among high school students [3, 4, 6]. This study expands the research by considering the general lifestyle habits and demographic information of high school students to explore their association with NSP using logistic regression. According to our survey results, the total prevalence of NSP was 23.7% among 4793 high school students. Demographic factors (including gender, grade and subject selection) and lifestyle factors (including sedentary time, poor posture, exercise habits, backpack weight and electronic device use habits) all had a great impact on the incidence of NSP.

The prevalence of NSP is higher among girls than among boys, which is consistent with previous studies [14, 15]. This finding may have several explanations. First, boys are more active in sports activities than girls [16], and appropriate exercise can relax and exercise the neck and shoulder muscles, thus relieving the symptoms of NSP [17]. Second, previous studies suggested that the physical strength and spinal load bearing capacity of females is weaker and that the pain threshold is different between men and women [15, 18, 19]. Third, women are more susceptible than men to negative emotions and are more sensitive to pain [20]. Therefore, female students are more susceptible to NSP.

In high school students, the prevalence of NSP in grade two and grade three students was significantly higher than that in grade one, possibly because the incidence of NSP is related to long-term neck and shoulder muscle strain. Compared with grade one students, grade two and grade three students generally accumulate more neck and shoulder strain, so the prevalence is higher. In addition, the prevalence of NSP among high school students who choose liberal arts majors is higher than that of science, partly because there were more girls majoring in liberal arts.

Today’s high school students are under heavy academic pressure and need to learn multiple courses at the same time. The textbooks and exercise books of various subjects have greatly increased the weight of the backpack. Our statistics show a negative correlation between backpack weight and the prevalence of NSP, which is consistent with previous studies [5, 21]. Studies have shown that to balance the load of the backpack, students will lean forward to change their center of gravity [22]. A heavy backpack can make the body lean forward; thus, spine flexion and pelvis forward tilt will worsen, and rectus abdominis activity will decrease [23], eventually increasing the risk of NSP [22].

In addition to backpack weight, poor posture also plays a role in the prevalence of NSP. The most common poor postures of high school students are sitting with crossed legs, walking with a hunchback, carrying a backpack on one shoulder, leaning the neck forward and rounding the shoulders. Crossing the legs can cause an uneven pressure distribution in the lumbar and thoracic vertebrae, which in the long term can put pressure on the spinal nerves, causing lower back pain [24, 25]. When carrying a backpack on one shoulder, the supporting shoulder is raised significantly higher than the other shoulder, which leads to scoliosis and increases the risk of NSP [21, 26]. Walking posture such as hunching the back, leaning the neck forward and rounding the shoulders can lead to long-term strain on the neck muscles, thus increasing the prevalence of NSP. Furthermore, the COVID-19 pandemic has significantly increased the time people spend using electronic devices and maintaining poor postures at home, eventually increasing the risk of NSP.

Lack of exercise in today’s Chinese adolescents is common [27]. Our statistics show a negative correlation between the increasing of exercise frequency and the prevalence of NSP. Regular physical exercise reduces the prevalence of NSP, likely because it strengthens the muscles of the shoulder, neck and lower back and reduces muscle strain caused by sedentary and poor posture [5]. In terms of exercise mode, moderate to vigorous physical activities help to reduce the incidence of NSP, which corresponds with the results of Pirnes KP et al. [28]. Moderate physical activity, such as slow walking, has no significant effect on reducing the prevalence of NSP. Due to the impact of the COVID-19 epidemic, outdoor activities have been greatly reduced, and the alternative to outdoor physical exercise is mostly moderate indoor exercise, which has no significant effect on relaxing neck and shoulder muscles and can increase the risk of NSP.

Electronic device usage is an important factor associated with the prevalence of NSP among high school students [29]. Our survey results show that long-term use of various electronic devices and poor posture are in direct proportion to the prevalence of NSP. When using a computer, the majority of respondents tended to lower their heads. Studies have shown that the mechanical requirements for the posterior neck muscles in the flexion cervical spine posture were 3–5 times higher than in the neutral position [7]. Bowing the head for a long time puts the muscles and ligaments of the posterior neck in a state of long-term traction, which can easily cause fatigue, injury and NSP; some studies have pointed out [30] that, compared with using standard desktop computers, using tablets and laptops results in greater neck flexion, bilateral shoulder elevation and upper trunk flexion.

Studies have shown that when using handheld electronic devices such as mobile phones and tablets, which are usually placed below the eyes, neck flexion is exacerbated [31–33]. In addition, using a mobile phone was associated with a significant increase in chest kyphosis, trunk tilt while standing and walking and lumbar lordosis [34], causing the neck muscles to bear a greater load compared with using a computer, and is more likely to cause muscle and ligament strain, resulting in NSP. In addition, due to the impact of COVID-19, the respondent group has also experienced a greatly increased length of time and frequency spent using various electronic devices, and the lack of activities to relax neck and shoulder muscles also increases the risk of NSP.

This study shows that sedentary time is also closely related to the prevalence of NSP. The prevalence of sedentary behavior is high among adolescents, and the school environment represents an important setting for the promotion of a sedentary lifestyle [35]. The results of our study show that most high school students have sedentary habits (sitting for 2 h). People who are often sedentary are more likely to have NSP. This higher risk may be because the most common sitting positions, such as those that include back bending, shoulder ptosis, and sitting for a long time, will cause compression of the spine, resulting in disc wear and overstrain injury of neck and lumbar back muscles, causing neck, shoulder, waist and back discomfort [36]. However, whether sitting for too long is a risk factor for NSP remains controversial [37]. In addition, the significant increase in sedentary time during the COVID-19 outbreak is a risk factor for the increased prevalence of NSP.

This was a cross-sectional survey conducted during the COVID-19 outbreak, and we found that the prevalence of NSP among high school students was 23.7%, which is significantly higher than previous 20.3% in Chinese high school students [38]. The explanation for this finding may be that as a result of the COVID-19 outbreak, students’ time spent at home for online classes increased significantly [39], the time and frequency of using electronic devices also increased significantly [40], and the time allotted for students’ to engage in physical exercise was significantly shortened [41]. Long-term online classes can easily cause students to increase their sedentary time and have poor posture [42]. All of these factors are risk factors for NSP, resulting in a significant increase in the prevalence of NSP among high school students during the COVID-19 outbreak.

### Strengths and limitations

The present study has its qualities and shortcomings. Previous studies on this topic were mostly conducted before the COVID-19. The lifestyle changes brought by COVID-19 to students will lead to the change of risk factors for NSP. In addition, most of the previous studies focused on college students, and few studies focused on the risk factors related to NSP in high school students during COVID-19. Our study provides an effective supplement in this regard. Limitations of this study include the finding that the prevalence of NSP increased during COVID-19, which may be affected by may be affected by errors in research methods, differences in NSP definition and other aspects. Besides, the determination of activity levels in our study is not based on objective measures but rather on subjective categorizations, which may introduce some level of subjectivity and potential inaccuracy in our results on activity levels. Additionally, this is a cross-sectional design, which limits our ability to infer causation between the exposure and outcomes. Outcomes were also assessed at one time point.

## Conclusion

In summary, the prevalence of NSP among high school students is 23.7%, which is closely correlated with gender, grade, subject selection, backpack weight, poor postures and the use of digital devices. In addition, due to the impact of COVID-19, online classes have greatly changed the lifestyles and learning methods of high school students, which are related to the increasing prevalence of NSP [7, 41, 43]. We recommend that the government and schools should focus on the influence of online course education and propose a more scientific and reasonable course arrangement [44, 45]. In addition, indoor and outdoor exercise should be encouraged, and more attention should be given to student’s participation in offline PE(physical education) activities, muscle relaxation activities during class breaks, and adjusting posture and time spent using electronic equipment [46]. By following the above measures, the risk of NSP in adolescents will be possibly reduced.

## Data Availability

The datasets used and analysed during the current study available from the corresponding author on reasonable request.

## Abbreviations

NSP: neck and shoulder pain
ORs: odds ratios
CIs: confidence intervals
